# Noncompletion and nonpublication of trials studying rare diseases: A cross-sectional analysis

**DOI:** 10.1371/journal.pmed.1002966

**Published:** 2019-11-21

**Authors:** Chris A. Rees, Natalie Pica, Michael C. Monuteaux, Florence T. Bourgeois

**Affiliations:** 1 Division of Emergency Medicine, Boston Children’s Hospital, Boston, Massachusetts, United States of America; 2 Department of Pediatrics, Harvard Medical School, Boston, Massachusetts, United States of America; 3 Department of Pediatrics, Children’s National Medical Center, The George Washington School of Medicine & Health Science, Washington, DC, United States of America; 4 Pediatric Therapeutics and Regulatory Science Initiative, Computational Health Informatics Program, Boston Children’s Hospital, Boston, Massachusetts, United States of America; Harvard University, UNITED STATES

## Abstract

**Background:**

Rare diseases affect as many as 60 million people in the United States and Europe. However, most rare diseases lack effective therapies and are in critical need of clinical research. Our objective was to determine the frequency of noncompletion and nonpublication of trials studying rare diseases.

**Methods and findings:**

We conducted a cross-sectional analysis of randomized clinical trials studying rare diseases as defined by the Genetic and Rare Disease Information Center database that were registered in ClinicalTrials.gov between January 1, 2010, and December 31, 2012, and completed or discontinued by December 31, 2014. Our main outcome measures were the frequency of trial noncompletion and, among completed studies, frequency of trial nonpublication at 2 and 4 years following trial completion. Reasons for discontinuation were extracted from the registry, and trial sponsors were contacted for additional information, as needed. Two independent investigators performed publication searches for each trial in PubMed, EMBASE, and GoogleScholar, allowing for a minimum of 45 months between trial completion and publication. When a publication could not be identified, trial sponsors were contacted to confirm publication status. The impact of funding source on trial noncompletion was assessed with multivariable logistic regression, and the effect on time to publication was examined with Cox proportional hazards regression. Control variables included intervention type, trial phase, masking, enrollment, and study population. We analyzed 659 rare disease trials accounting for 70,305 enrolled patients. Industry was the primary funder for 327 trials (49.6%) and academic institutions for 184 trials (27.9%). There were 79 trials (12.0%) focused on pediatric populations. A total of 199 trials (30.2%) were discontinued. Lack of patient accrual (*n* = 64, 32.1%) and informative termination (*n* = 41, 20.6%) were the most common reasons for trial noncompletion. Among completed trials, 306 (66.5%) remained unpublished at 2 years and 142 (31.5%) at 4 years. In multivariable analyses, industry-funded trials were less likely to be discontinued than trials funded by healthcare centers (odds ratio [OR] 2.42; 95% confidence interval [CI] 1.34–4.39, *P* = 0.003). We found no significant association between funding source and time to publication. A total of 18,148 patients were enrolled in trials that were discontinued or unpublished 4 years after completion. A potential limitation of our study is that certain interventional trials for rare diseases may not have been registered in ClinicalTrials.gov, in particular Phase 0 and Phase I trials, which are not required to be registered.

**Conclusions:**

In this study, over half of clinical trials initiated for rare diseases were either discontinued or not published 4 years after completion, resulting in large numbers of patients with rare diseases exposed to interventions that did not lead to informative findings. Concerted efforts are needed to ensure that participation of patients in rare disease trials advances scientific knowledge and treatments for rare diseases.

## Abstract 2

In this cross-sectional study, Florence Bourgeois and colleagues investigate the frequency of and reasons for discontinuation of clinical trials studying rare diseases.

## Author summary

**Why was this study done?:**

- Rare diseases are a group of 7,000 conditions affecting approximately 60 million people in the US and Europe.
- Many rare diseases begin in childhood and are chronic conditions but lack therapeutic options because of the challenges of conducting trials in these populations.
- Clinical trial activities for rare diseases, including rates of trial completion and publication, have not been well defined.

**What did the researchers do and find?:**

- We analyzed rare disease trials registered in a clinical trial registry from 2010 to 2012 and determined the frequencies of trial noncompletion and nonpublication through 2018.
- Among 659 randomized controlled trials, 199 (30.2%) were discontinued, most often because of difficulties with patient accrual.
- There were 306 (66.5%) trials that were not published 2 years after completion and 142 (31.5%) that remained unpublished 4 years after trial completion.

**What do these findings mean?:**

- In our study, over half of randomized controlled trials studying rare diseases were not completed or were left unpublished 4 years after trial completion, highlighting considerable inefficiencies in research activities for these conditions.
- Increased focus on establishing global initiatives and networks to coordinate recruitment efforts may be needed, as well as additional mechanisms to ensure timely dissemination of research findings.

## Introduction

Rare diseases individually occur in fewer than 1 in 2,000 people but collectively affect almost 6% of people at some point during their lives [1]. There are approximately 7,000 rare diseases and 60 million people in the US and Europe who suffer from these conditions [1,2]. Many rare diseases are chronic and life threatening. Since the majority are genetic in origin, three-quarters of rare diseases also affect children [3]. Examples of rare diseases include cystic fibrosis, Huntington disease, and Duchenne muscular dystrophy, as well as acquired conditions such as mesothelioma and botulism.

Because of a number of regulatory and financial incentives promoting drug development for rare diseases, there has been a substantial increase in available therapies for rare conditions over the past 30 years [4]. However, less than 10% of rare diseases have an available therapy, and as few as 22% of these conditions have been studied in drug trials [5,6]. Challenges in performing rare disease trials include small and geographically dispersed patient populations, inaccurate diagnostic and effect measurements related to clinical heterogeneity, lack of validated clinical endpoints, and limited disease expertise in the medical community [7,8]. Previous studies examining rare disease trials have shown that, compared to trials for nonrare diseases, rare disease trials tend to enroll fewer participants, use single-arm or nonrandomized designs, and measure surrogate endpoints as opposed to overall survival [9].

Given the critical need for therapeutic development for rare diseases and the small patient populations available to generate robust clinical evidence, it is essential that clinical trials initiated for these conditions are completed and trial results disseminated in a timely fashion. To elucidate the epidemiology of randomized clinical trials studying rare diseases, our objective was to determine the frequency of noncompletion and nonpublication of rare disease trials and identify factors associated with these outcomes.

## Methods

### Data source

We conducted a cross-sectional analysis of randomized clinical trials focusing on rare diseases registered in ClinicalTrials.gov. The ClinicalTrials.gov query and data download were performed on a single day (March 3, 2017) to account for ongoing updates to database records. The Institutional Review Board at Boston Children’s Hospital exempted this study from review because it did not represent human participant research. We followed a prospective protocol for data extraction, classification, and analysis (S1 Text).

We reviewed randomized clinical trials that were registered between January 1, 2010, and December 31, 2012, and completed or discontinued by December 31, 2014. This timeframe was chosen to allow sufficient time for follow-up to trial publication. Discontinued trials were defined as those with a status of “terminated,” “withdrawn,” or “suspended” [10,11]. We excluded trials registered more than 60 days after the start date to avoid bias related to decisions made after initial trial findings become available [10,12]. Rare disease trials were identified using the ClinicalTrials.gov study topic of “Rare Diseases.” Each trial entry was then manually reviewed by two of the authors (NP and CAR) to confirm the trial focused on a rare disease listed in the Genetic and Rare Disease (GARD) Information Center database, a resource compiled by the National Institutes of Health’s National Center for Advancing Translational Sciences and the National Human Genome Research Institute [13]. Rare diseases were grouped according to categories provided by GARD [14]. If trials listed multiple conditions, more than half of the conditions needed to meet our definition of a rare disease in order for the trial to be included. Infectious disease trials were excluded because these diseases are often rare in developed nations but represent a large disease burden globally and therefore face different considerations around trial conduct compared to trials for noninfectious rare diseases.

### Definitions and data characterization

We used the Glossary of Common Site Terms and ClinicalTrials.gov Protocol Data Element Definitions to define variables [10]. Primary funders were defined as the organization listed as the sponsor of a study. In cases when more than one sponsor was listed, the lead sponsor was designated as the primary funding source [15]. Funding sources in ClinicalTrials.gov are classified as industry, US government (National Institutes of Health and other US federal agencies), or “other,” which includes foundations and research alliances. We created new funding variables for academic institutions and healthcare centers by reviewing all funders listed as “other.” In addition, we added non–US government funding sources to US government to create our final funding sources of industry, academic institutions, healthcare centers, government, and other. We defined pediatric trials as those that exclusively studied children 0–17 years of age or those that also included adults but in which the midpoint of the age eligibility range was <18 years [16]. Based on trial locations listed in ClinicalTrials.gov, we classified trials as conducted entirely in the US, entirely outside of the US, or in both the US and other countries. We defined time to publication as the interval between the “primary completion date” and the date the publication appeared in print or as an electronic article, whichever occurred first. In cases when the primary completion date was missing (*n* = 31), we used the listed “completion date.”

Reasons for trial noncompletion were determined based on data provided in ClinicalTrials.gov records. If the reason for noncompletion was not listed or unclear, we contacted study investigators and sponsors to obtain additional information. Reasons for noncompletion included insufficient patient accrual, informative termination (e.g., safety or toxicity concerns or interim study results indicating benefit or futility), company/business decisions, funding issues, conduct problems (e.g., insufficient study drug or technical problems with trial rollout), regulatory issues (e.g., difficulty obtaining approval from institutional review boards or other regulatory bodies), principal investigator departure, or none reported.

### Publication search

We reviewed all trial entries in ClinicalTrials.gov to search for publications added via the national clinical trial (NCT) identifier number. For trials without a listed published article in the trial entry, Medline was searched via PubMed independently by two investigators (NP and CAR) using NCT number, trial title, author names, disease studied, intervention, study dates, and features of the study design. The search protocol was repeated in both EMBASE and GoogleScholar if no publication was identified in Medline. For industry-sponsored trials, we also searched company websites for publication information, as needed.

If a trial could not be linked to a published article, we contacted study investigators and sponsors to inquire about trial publication status. We obtained email addresses from ClinicalTrials.gov entries, previous publications by the investigators, and online searches for contact information. A standard email was sent to each investigator with an additional email sent 2 weeks later if there was no response [11]. For trials that listed only a sponsoring company, we contacted responsible individuals by email, online forms, or telephone as per company instructions.

We reviewed identified publications in full to ensure a match with the respective Clinicaltrials.gov entry and to verify that it was a peer-reviewed article describing trial findings. We classified trials as unpublished if we could not identify a corresponding published article or if trial investigators reported that the trial was not published. A final search for trial publications was conducted on September 27, 2018, allowing a minimum of 45 months between trial completion and publication.

### Statistical analyses

We used chi-squared tests and nonparametric equality of median tests to compare categorical and continuous characteristics, respectively, of completed and noncompleted trials. For completed trials, we compared characteristics of published and nonpublished trials at 2 and 4 years after trial completion. Kaplan-Meier curves were used to illustrate time to publication for completed trials, stratified by funding source. The impact of funding source on trial noncompletion was assessed with a multivariable logistic regression model with noncompletion status as the dependent variable and funding source, modeled as a set of dummy variables with industry set as the referent, as the independent variable. Effect estimates were reported as adjusted odds ratios (aORs) and 95% confidence intervals (CIs). The impact of funding source on time to publication was examined with a multivariable Cox proportional hazards model with time to publication (starting from the completion date) as the dependent variable and funding source (modeled as described above) as the independent variable. Observations were censored if not published by the end of the follow-up period (September 27, 2018). Control variables for both models were prespecified and consisted of key elements of trial design, including intervention type, trial phase, masking, and study population. Trial sample size (modeled as a piecewise linear spline with knots at the 25th, 50th, and 75th percentiles) was not included in the model examining noncompletion (since these trials halt enrollment early) but was included in the model assessing time to publication. We conducted post hoc pairwise comparisons of the five funding sources with a Bonferroni correction (with 10 pairwise comparisons, the Bonferroni-adjusted alpha level for these tests was 0.05/10 = 0.005) to determine funding sources that were significantly associated with trial noncompletion and time to publication. In addition, a post hoc multivariable analysis was performed with discontinuation because of poor patient accrual (compared to trials that were completed) as the dependent variable and funding source as the independent variable (modeled as a binary variable of industry or nonindustry), using the same control variables as in the primary analyses. Statistical significance was prespecified at a *P* value of <0.05. All analyses were conducted in Stata/SE version 14.1 (StataCorp, College Station, TX, USA).

## Results

**Fig 1:**
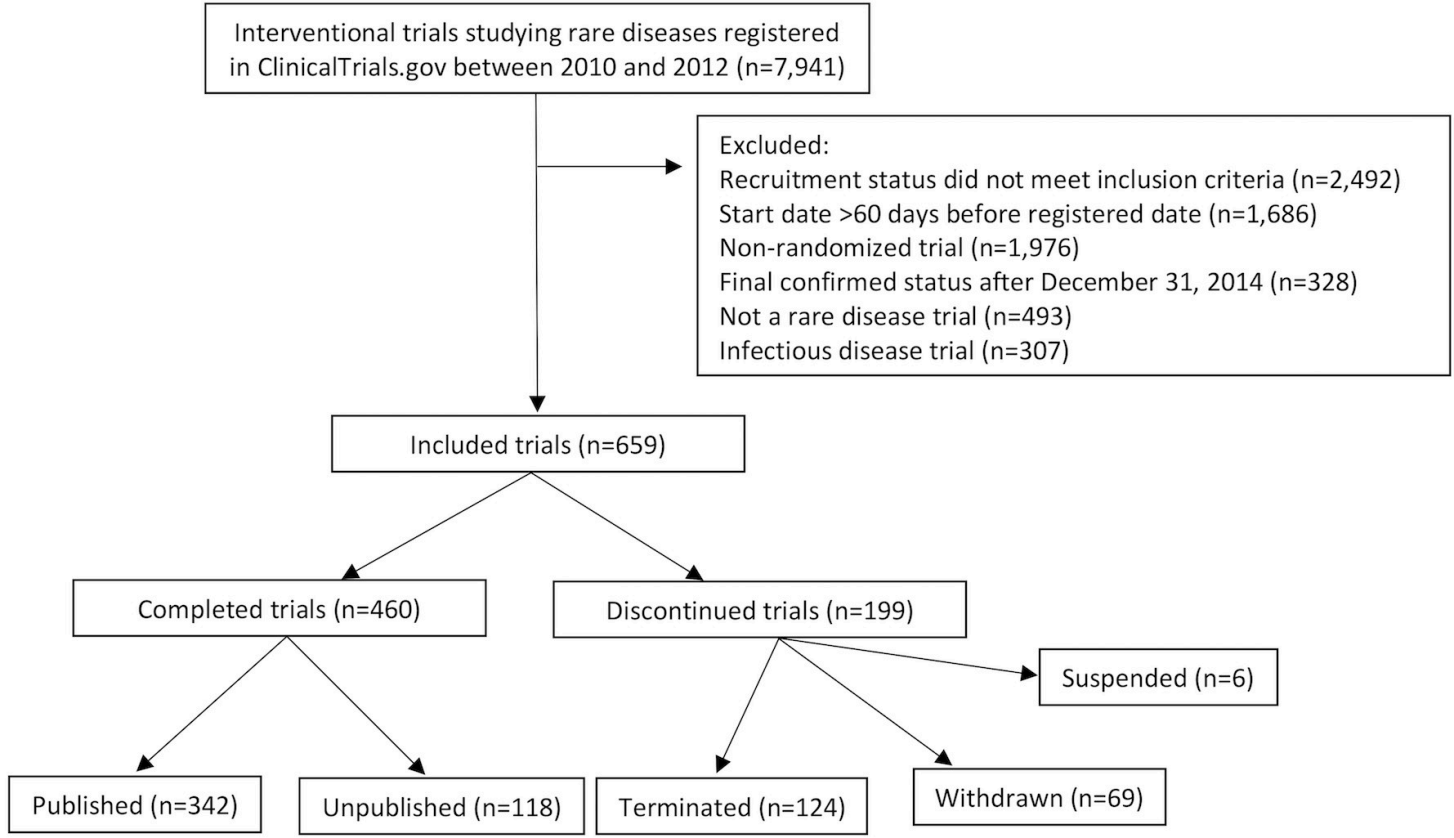
Selection of rare disease trials.

We identified 659 randomized clinical trials studying rare diseases and meeting our inclusion criteria (Fig 1). A total of 70,305 patients were enrolled in these trials. The most common rare disease categories studied were cancers (*n* = 173, 26.4%), lung diseases (*n* = 81, 12.3%), and nervous system diseases (*n* = 72, 10.9%) (Table 1). Industry was the primary funder for 329 trials (50.0%) and academic institutions the primary funder for 198 trials (30.0%) (Table 2). The majority of trials (*n* = 542, 82.3%) studied drugs or biologics. There were 357 trials (54.2%) that were double blind, 256 (38.9%) that employed an open-label design, and 46 (7.0%) that were single blind. Among completed trials, the median number of enrolled participants was 61 (interquartile range [IQR] 30–124), with 74.5% (*n* = 491) of completed trials enrolling fewer than 100 patients. Only 79 trials (12.0%) focused on pediatric populations. Trial locations were entirely in the US for 29.9% (*n* = 197) of trials, whereas 42.2% (*n* = 278) of trials were conducted entirely in other countries, and 20.0% (*n* = 132) were conducted in both the US and other countries (7.9% [*n* = 52] did not list location).

**Table 1:** Disease categories for 659 randomized clinical trials studying rare diseases.

Disease Category	*n* (%)
Cancers	173 (26.4)
Lung diseases	81 (12.3)
Nervous system diseases	72 (10.9)
Blood diseases	64 (9.7)
Heart diseases	47 (7.1)
Congenital and genetic diseases	30 (4.6)
Digestive diseases	26 (3.9)
Eye diseases	26 (3.9)
Skin diseases	21 (3.2)
Autoimmune/autoinflammatory diseases	19 (2.9)
Musculoskeletal diseases	19 (2.9)
Endocrine diseases	18 (2.7)
Female reproductive diseases	18 (2.7)
Nutritional diseases	11 (1.7)
Metabolic disorders	10 (1.5)
Kidney and urinary diseases	8 (1.2)
Connective tissue diseases	6 (0.9)
Hereditary cancer syndromes	4 (0.6)
Myelodysplastic syndromes	4 (0.6)
Mouth disorders	2 (0.3)

**Table 2:** Characteristics of completed and discontinued trials for rare diseases.

Characteristics	All Trials (*n* = 659), *n* (%)	Completed Trials (*n* = 460), *n* (%)	Discontinued Trials (*n* = 199), *n* (%)	*P* Value
Funding source				0.001
Industry	329 (50.0)	249 (54.1)	80 (40.2)	
Academic institution	198 (30.0)	136 (29.6)	62 (31.2)	
Government	24 (3.6)	16 (3.5)	8 (4.0)	
Healthcare center	74 (11.2)	42 (9.1)	32 (16.1)	
Other	34 (5.2)	17 (3.7)	17 (8.5)	
Intervention				0.612
Drug/biologic	542 (82.3)	378 (82.2)	164 (82.4)	
Behavioral	12 (1.8)	9 (1.9)	3 (1.5)	
Device/procedure	59 (9.0)	39 (8.5)	20 (10.1)	
Dietary supplement	19 (2.9)	12 (2.6)	7 (3.5)	
Other	27 (4.1)	22 (4.8)	5 (2.5)	
Trial phase^a^				0.041
I	79 (12.0)	67 (14.6)	12 (6.0)	
II	256 (38.9)	176 (38.3)	80 (40.2)	
III	185 (28.1)	123 (26.7)	62 (31.2)	
IV	49 (7.4)	34 (7.4)	15 (7.5)	
Not specified	90 (13.7)	60 (13.0)	30 (15.1)	
Masking				0.060
Open label	256 (38.9)	167 (36.3)	89 (44.7)	
Single blind	46 (7.0)	37 (8.0)	9 (4.5)	
Double blind	357 (54.2)	256 (55.6)	101 (50.7)	
Enrollment				N/A^b^
Median enrollment (IQR)	40 (14–100)	61 (30–124)	5 (0–28)	
Study population				0.970
Adult	580 (88.0)	405 (88.0)	175 (87.9)	
Pediatric	79 (12.0)	55 (12.0)	24 (12.1)	

^a^Thirty-two trials listed as Phase I/II were categorized as Phase II, and 26 trials listed as Phase II/III were categorized as Phase III.

^b^Discontinued trials halt patient enrollment early, and therefore, *P* values assessing difference in enrollment were not calculated.

Abbreviation: IQR, interquartile range.

### Noncompletion of trials

Of the 659 trials, 30.2% (*n* = 199) were discontinued. The most common reasons for trial noncompletion were insufficient patient accrual (31.2%, *n* = 62), informative termination (22.6%, *n* = 45), and company/business decision (16.0%, *n* = 32) (Table 3). Participant enrollment was underway for 65.3% (*n* = 130) of trials at the time of study discontinuation, with 6,349 patients enrolled in trials that were discontinued. More than half of these patients (55.9%, *n* = 3,548) were enrolled in trials discontinued based on informative reasons, such as emerging safety and efficacy data related to the intervention.

**Table 3:** Reasons for noncompletion of rare disease trials.

Reason for Noncompletion^a^	Trials, *n* (%)	Patients Enrolled, *n* (%)
Patient accrual	62 (31.2)	924 (14.5)
Informative termination	45 (22.6)	3,548 (55.9)
Company/business decision	32 (16.0)	1,054 (16.6)
None reported	22 (11.1)	139 (2.2)
Funding issue	20 (10.1)	315 (5.0)
Conduct problems	15 (7.5)	356 (5.6)
Regulatory issue	2 (1.0)	0 (0.0)
Principal investigator departure	1 (0.5)	13 (0.2)
Total	199 (100.0)	6,349 (100.0)

^a^Eight trials listed a secondary reason for discontinuation, including patient accrual (*n* = 3), funding issue (*n* = 2), company/business decision (*n* = 1), informative termination (*n* = 1), and conduct problem (*n* = 1).

In univariate analysis, funding source and trial phase were significantly associated with trial noncompletion (Table 2). Among trials with industry funding, 24.3% (80/329) were discontinued, compared with 31.3% (62/198) of trials with academic funding, 33.3% (8/24) of trials with government funding, and 43.2% (32/74) of trials funded by healthcare centers. Funding source remained associated with trial discontinuation in multivariable analysis, with trials funded by industry significantly less likely to be discontinued than trials funded by healthcare centers (odds ratio [OR] 2.42; 95% CI 1.34–4.39, *P* = 0.003) or other funding sources (OR 2.79; 95% CI 1.33–5.86, *P* = 0.007) (Table 4). Following pairwise comparisons with Bonferroni correction, only the difference between industry- and healthcare center–funded trials remained significant. Trials funded by industry were significantly less likely to be discontinued because of poor patient accrual than trials funded by nonindustry (OR 0.22; 95% CI 0.11–0.44, *P* < 0.001).

**Table 4:** Multivariable analysis for impact of funding source on noncompletion and time to publication of trials for rare diseases.

	Trial Noncompletion			Time to Trial Publication		
Trial Characteristics	Odds Ratio	95% CI	*P* Value	Hazard Ratio	95% CI	*P* Value
Funding source						
Industry	Referent				Referent	
Academic institution	1.49	0.95–2.33	0.084	1.16	0.86–1.57	0.339
Government	1.65	0.66–4.14	0.286	1.92	1.06–3.49	0.032^b^
Healthcare center	2.42	1.34–4.39	0.003^a^	1.51	1.00–2.27	0.050
Other	2.79	1.33–5.86	0.007	0.99	0.54–1.77	0.937
Intervention						
Drug/biologic	Referent			Referent		
Behavioral	0.61	0.15–2.53	0.492	1.27	0.52–3.07	0.597
Device/procedure	0.83	0.43–1.62	0.582	1.28	0.83–1.97	0.272
Dietary supplement	1.05	0.38–2.86	0.925	1.12	0.57–2.22	0.738
Other	0.35	0.11–1.04	0.060	1.60	0.88–2.89	0.120
Trial phase^c^						
I	0.46	0.18–1.14	0.094	0.70	0.39–1.25	0.225
II	1.06	0.53–2.13	0.870	1.24	0.77–1.99	0.373
III	1.27	0.62–2.60	0.511	1.64	1.03–2.63	0.039 ^b^
IV	Referent			Referent		
Unknown	1.19	0.53–2.67	0.673	1.41	0.81–2.42	0.221
Masking						
Open label	Referent			Referent		
Single blind	0.47	0.21–1.06	0.068	1.29	0.82–2.02	0.267
Double blind	0.72	0.49–1.05	0.092	1.08	0.82–1.38	0.658
Enrollment						
0–14 participants	N/A^d^	N/A		1.07	0.98–1.17	0.114
15–40 participants	N/A	N/A		1.00	0.98–1.02	0.938
41–100 participants	N/A	N/A		1.00	1.00–1.01	0.562
≥100 participants	N/A	N/A		1.00	1.00–1.00	0.033^b^
Study population						
Adult	Referent			Referent		
Pediatric	0.91	0.53–1.56	0.719	0.83	0.59–1.18	0.311

^a^Comparison of industry-funded trials to healthcare center–funded trials significant after Bonferroni correction. None of the other 9 pairwise comparisons were significant after Bonferroni correction.

^b^Neither government-funded trials, Phase III trials, or higher enrollment remained significantly associated with time to publication following Bonferroni correction.

^c^Thirty-two trials listed as Phase I/II were categorized as Phase II, and 26 trials listed as Phase II/III were categorized as Phase III.

^d^Discontinued trials halt patient enrollment early, and therefore, this variable was not included in models examining trial noncompletion.

### Nonpublication of trials

We identified 342 publications, 114 (33.3%) in the ClinicalTrials.gov registry, 221 (64.6%) through searches in bibliographic databases, and 7 (2.1%) through correspondence with investigators and sponsors. The median follow-up since trial completion was 66 months (minimum of 45 months). Twenty-six (22%) of the unpublished trials had results posted in the ClinicalTrials.gov registry.

**Fig 2:**
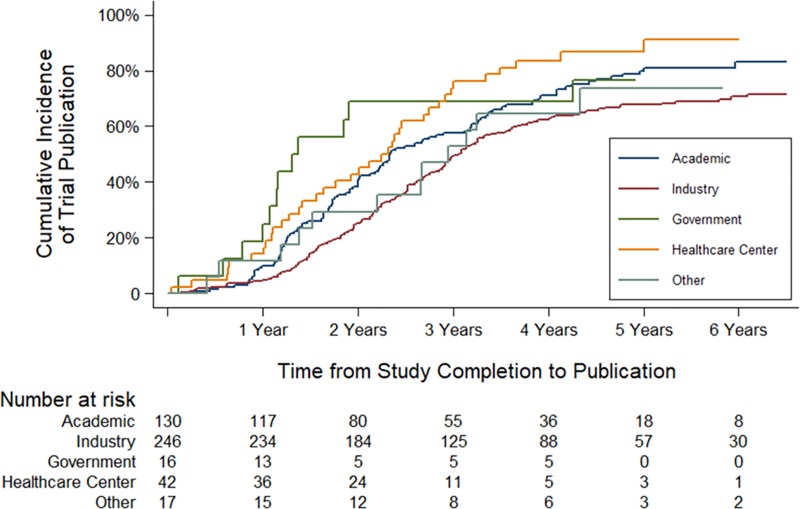
Time to publication of rare disease trials. Cumulative incidence of publication for rare disease trials using Kaplan-Meier methods. Of the trials, 33.5% were published by 2 years and 68.5% by 4 years, with a median time to publication of 2.2 years.

The median time to publication was 26 months (IQR 17–37), with 66.5% (306/460) of trials unpublished at 2 years and 31.5% (142/451) at 4 years (Table 5 and Fig 2). This corresponded to 36,581 participants in trials that were unpublished at 2 years and 11,799 participants in trials that were unpublished at 4 years. Nonpublication of trials at 2 years was associated with funding source and trial phase in univariate analyses (Table 5). Among completed trials funded by industry, 74.3% (185/249) were unpublished at 2 years, compared with 58.8% (80/136) of trials funded by academic institutions, 31.2% (5/16) of government-funded trials, and 57.1% (24/42) of trials funded by healthcare centers. In multivariable Cox proportional hazards models, government-funded trials were published faster than industry-funded trials (hazard ratio [HR] 1.92; 95% CI 1.06–3.49, *P* = 0.032), though this did not remain significant in pairwise testing of funding sources after Bonferroni correction.

**Table 5:** Characteristics of published and unpublished rare disease trials that were completed^a^.

Trial Characteristics	Unpublished at 2 Years, (*n* = 306) *n* (%)	Published at 2 Years, (*n* = 154) *n* (%)	*P* Value	Unpublished at 4 Years, (*n* = 142^a^) *n* (%)	Published at 4 Years, (*n* = 309) *n* (%)	*P* Value
Funding source			<0.001			0.032
Industry	185 (60.5)	64 (41.6)		89 (62.7)	155 (50.1)	
Academic institution	80 (26.1)	56 (36.4)		37 (26.1)	97 (31.4)	
Government	5 (1.6)	11 (7.1)		5 (3.5)	11 (3.6)	
Healthcare center	24 (7.8)	18 (11.7)		5 (3.5)	35 (11.3)	
Other	12 (4.0)	5 (3.2)		6 (4.2)	11 (3.6)	
Intervention			0.221^b^			0.021^b^
Drug/biologic	257 (84.0)	121 (78.6)		129 (90.9)	241 (78.0)	
Behavioral	5 (1.6)	4 (2.6)		1 (0.7)	8 (2.6)	
Device/procedure	25 (8.2)	14 (9.1)		6 (4.2)	32 (10.4)	
Dietary supplement	9 (2.9)	3 (1.9)		3 (2.1)	9 (2.9)	
Other	10 (3.3)	12 (7.8)		3 (2.1)	19 (6.1)	
Trial phase^b^			0.036			<0.001
I	53 (17.3)	14 (9.1)		38 (26.8)	29 (9.4)	
II	121 (39.6)	55 (35.7)		56 (39.5)	116 (37.5)	
III	74 (24.2)	49 (31.8)		28 (19.7)	93 (30.1)	
IV	24 (7.8)	10 (6.5)		10 (7.0)	22 (7.1)	
Unknown	34 (11.1)	26 (16.9)		10 (7.0)	49 (15.9)	
Masking			0.106			0.111
Open label	116 (37.9)	51 (33.1)		53 (37.3)	111 (35.9)	
Single blind	19 (6.2)	18 (11.7)		6 (4.2)	31 (10.0)	
Double blind	171 (55.9)	85 (55.2)		83 (58.5)	167 (54.1)	
Enrollment^c^	59 (26–120)	68 (30–130)	0.199^d^	47 (20–88)	68 (32–137)	0.001^d^
Study population			0.179			0.999
Adult	265 (86.6)	140 (90.9)		125 (88.0)	272 (88.0)	
Pediatric	41 (13.4)	14 (9.1)		17 (12.0)	37 (12.0)	

^a^There were 9 trials that did not have 4 years of follow-up and were excluded.

^b^Thirty-two trials listed as Phase I/II were categorized as Phase II, and 26 trials listed as Phase II/III were categorized as Phase III.

^c^Reported in median and (interquartile range).

^d^Nonparametric equality-of-medians test.

### Pediatric rare disease trials

Of the 79 pediatric trials for rare diseases, lung diseases (21.5%, *n* = 17) and nervous system diseases (13.9%, *n* = 11) were the most commonly studied disease categories. The median enrollment was 26 (IQR 12–60). Children 0 to 6 years of age were the most commonly enrolled population (40.0%, *n* = 30). The rate of trial discontinuation for pediatric trials was 30.4% (24/79), which was similar to the discontinuation rate for trials focused on adults (30.2% [175/580], *P* = 0.969). Pediatric trials were discontinued most often because of insufficient patient accrual (41.7%, *n* = 33) and funding issues (16.5%, *n* = 13). Completed pediatric trials were unpublished at similar rates compared to adult trials (23.6% [13/55] versus 25.9% [105/405], respectively, *P* = 0.715), with 74.6% (41/55) unpublished at 2 years and 32.7% (18/55) at 4 years after completion. A total of 354 pediatric patients were enrolled in trials that were discontinued and 741 in trials that were completed but not published.

## Discussion

In this analysis of rare disease trials, over half the trials were not completed or were left unpublished 4 years after trial completion. These findings highlight considerable inefficiencies in research activities for rare diseases. Difficulties with participant accrual accounted for about a third of trial noncompletions, whereas 22% occurred for informative reasons, such as results from interim analyses. Among completed trials, almost a third were not published in the peer-reviewed literature after 4 years, and there were also substantial delays among trials that were published, with greater than two-thirds unpublished at 2 years after trial completion. Funding source was a determinant of trial completion, with trials funded by industry less likely to be discontinued than trials funded by healthcare centers. In all, more than 18,000 participants were enrolled in trials that were not completed or remained unpublished 4 years after completion.

Insufficient patient accrual was the most common reason for trial noncompletion. This finding is in line with prior studies assessing discontinuation across trials for various nonrare conditions and patient populations [10,11,17,18]. Although analyses have demonstrated conflicting findings on the role of funding source on trial discontinuation, our results were consistent with prior studies indicating that industry-funded trials are less likely to be discontinued and also less likely to be discontinued specifically because of poor patient accrual [10,19–21]. This may be related to greater resources and infrastructure among industry sponsors for supporting participant recruitment and retention. Informative termination was also a common reason for trial noncompletion, consistent with prior reports [10,11,18]. Discontinuation of certain trials related to findings in interim analysis is to be expected, and data and safety monitoring boards are specifically charged with making recommendations on the continuation or termination of a trial based on scheduled reviews. In these cases, publication of trial results to the point of discontinuation remains critical to inform future research programs and clinical care.

Nearly one in three trials for rare diseases were completed, but the results were not available in the peer-reviewed literature 4 years after trial completion. This rate of nonpublication aligns with previous studies evaluating nonpublication rates across a number of other cohorts, including surgical trials, vaccine trials, trials of neurodegenerative diseases, pediatric trials, and trials with specific types of funding sources [11,18, 21–24]. Trial nonpublication rates among these studies ranged from 25% to 35%, with median follow-up periods of 2 to 5 years. Studies examining factors associated with nonpublication have identified industry funding, early trial phase, and certain design features (e.g., single-blind status) as significant predictors of nonpublication [10–12,21,23].

Children may be particularly vulnerable to the challenges of conducting clinical trials for rare diseases. However, we found similar rates of noncompletion and nonpublication among pediatric and adult studies. The pediatric trial discontinuation rate of 30% in our study is comparable with prior studies demonstrating noncompletion among 18% to 40% of trials in pediatric populations [10,20,25,26]. The nonpublication rate of 24% for rare disease trials was slightly lower than previously reported rates of 30% to 37% among various cohorts of pediatric trials and may reflect improvements in this practice over time [10,20,25,26].

### Implications for clinicians and policymakers

It is estimated that 94% of rare diseases continue to lack an approved therapy [6]. Because of the scarcity of treatment options and low quality of evidence for many rare disease therapies, 35 countries have implemented regulations and policies to increase trial activity for rare diseases [4]. In 2011, the International Rare Diseases Research Consortium (IRDiRC) was launched and set a goal to be able to diagnose most rare diseases and to develop 200 new therapies by 2020 [6]. The goal for drug development was achieved in 2017, 3 years ahead of schedule, in large part because of a surge in public-sector research initiatives aimed at addressing rare diseases, improved public awareness about rare diseases, and increased engagement in partnerships between the scientific community, industry, patients, and policymakers. Building on this momentum, for the coming decade, the IRDiRC has called for a globally coordinated diagnostic and research initiative to shorten times to diagnosis and to further accelerate the rate of drug development, especially for rare diseases without existing therapeutic options [6].

The high rate of trial discontinuation related to inadequate patient enrollment underscores the importance of establishing global initiatives and networks to coordinate recruitment efforts. In designing research programs, given the added challenges of accruing participants when patient populations are limited and geographically dispersed, proactive collaboration with rare disease experts and patient advocacy groups should be prioritized to facilitate robust enrollment forecasting and maximize trial communication to potential participants. It is also critical that stakeholders adopt a disease-focused approach to ensure patient participation in the most promising trials, as opposed to diffuse enrollment based on sponsor needs. These efforts should be designed with an eye to preventing trial initiation—and futile patient enrollment—until there is reasonable certainty that the required sample size can be achieved. Scientific review boards might play a role in this process by ensuring that only studies that are deemed feasible based on robust enrollment estimates are allowed to begin patient recruitment.

Timely publication of trial results, particularly for diseases with a scarcity of treatment options, is imperative. Beyond the scientific mandate for knowledge dissemination, trial investigators and sponsors have an ethical obligation to study participants to maximize the benefit derived from their participation in research studies [27]. Policies and initiatives such as the Declaration of Helsinki, the US Federal Policy for the Protection of Human Subjects, and the Restoring Invisible and Abandoned Trials initiative, emphasize investigators’ duty to ensure timely publication of accurate and complete trial results [28–30]. ClinicalTrials.gov has supported the public posting of trial results since 2008 [31], but investigators must also publish their results in peer-reviewed journals. The peer-review process ensures rigorous and accurate analyses, reduces study biases, and contextualizes study findings [32,33]. Moreover, peer-reviewed publications provide more accessible study results than do registries for clinicians and the scientific community, thus further supporting the dissemination and application of clinical evidence. To increase timely availability of research data, investigators can complement the peer-review process with preprint services such as medRxiv, which aim to increase the accessibility and dissemination of scientific findings [34].

Completion and reporting of pediatric trials for rare diseases is particularly pressing [35]. Despite a number of economic incentives to increase pediatric drug research and development of therapies for pediatric rare diseases [36,37], a recent review reported that only 35% of 133 approved medicinal products for rare diseases were approved for use in children in the US and 47% in the European Union [38]. Policies have been implemented in the US and the EU to promote pediatric research and drug development [39]. These policies have led to increases in the number of pediatric drug trials and the number of drug labels that include pediatric prescribing information [40,41]. However, many drugs are exempted from pediatric study requirements, whereas others are associated with pediatric trials that are delayed until many years after a drug has gained market approval for use in adults [35,42]. In the US, the Rare Pediatric Disease Priority Review Voucher Program was implemented in 2012 to incentivize specifically the development of novel therapies for pediatric rare diseases. Given the recent initiation of the program, it remains unclear what the long-term impact of this program will be. However, a recent analysis indicates that the program has successfully increased the rate of product progression from Phase I to Phase II clinical trials but has not increased the rate of new pediatric drug development overall [37].

### Strengths and limitations

Because of a number of policies mandating trial preregistration, ClinicalTrials.gov has become the largest single trial registry and provides the most comprehensive data on trials for rare diseases worldwide [43–45]. Nonetheless, it is possible that certain interventional trials for rare diseases were conducted during the study period and not registered in the registry, in particular Phase 0 and Phase I trials, which are not required to be registered, or trials conducted entirely in non-US countries. However, our results indicate that a large number of trials conducted outside of the US are registered in ClinicalTrials.gov, which is consistent with prior reports [46]. Furthermore, it is possible that we were unable to identify all published articles resulting from completed trials. To minimize this, we employed a rigorous search strategy consisting of a standardized protocol, publication searches conducted independently by two investigators, and direct queries to investigators and sponsors, making the possibility of missed publications unlikely. In addition, we allowed for a long follow-up period—a minimum of 45 months between trial completion and publication—to ensure studies that were slow to be published would be captured. This approach also presents a limitation, however, because practices for trials registered since 2012 may have evolved, and ongoing assessments of the conduct and result dissemination of rare disease trials are needed. We also did not evaluate rates of publication of noncompleted trials, and additional research is required to further assess the dissemination of results from such trials. In particular, the publication of trials terminated for informative reasons should be assessed in order to ensure results from these trials are made available up through the point of discontinuation. Finally, we relied on trial information provided by investigators in the registry, including the status of trials, and were not able to independently verify these data elements.

## Conclusions

More than half of randomized controlled trials studying rare diseases were either discontinued or remained unpublished 4 years after trial completion, resulting in large numbers of patients with rare diseases exposed to interventions that did not lead to informative findings. High rates of insufficient patient accrual highlight the importance of establishing global initiatives and networks to coordinate patient enrollment efforts in rare disease trials. Concerted efforts are needed to ensure that participation of all patients in rare disease trials advances scientific knowledge and treatments for rare diseases.

## Supporting information

S1 STROBE Checklist STROBE statement—Checklist of items that should be included in reports of observational studies. STROBE, Strengthening the Reporting of Observational studies in Epidemiology.
(DOCX)

S1 Text Prospective protocol for data extraction, classification, and analysis. (DOCX)

## Abbreviations

aOR: adjusted odds ratio
CI: confidence interval
GARD: Genetic and Rare Disease
HR: hazard ratio
IQR: interquartile range
IRDiRC: International Rare Diseases Research Consortium
NCT: national clinical trial
OR: odds ratio.
