# Supplementary material for: Noncompletion and nonpublication of trials studying rare diseases: A cross-sectional analysis
Source: PLoS Med. 2019 Nov 21;16(11):e1002966. doi: 10.1371/journal.pmed.1002966 (PMC6871779; doi:10.1371/journal.pmed.1002966)
Supplement: S1 Text — (DOCX) [file pmed.1002966.s002.docx]

**Protocol: Non-completion and non-publication of trials studying rare diseases**

*Finalized: June 25, 2018*

**Primary outcomes:**

- Prevalence of trial discontinuation
- Prevalence of trial non-publication
- Time to trial publication

**Secondary outcomes:**

- Reasons for discontinuation
- Impact of funding source on trial discontinuation and time to publication
- Differences in completion and publication between adult and pediatric trials (birth to 17)

**Trial selection and data extraction from ClinicalTrials.gov:**

- Trials categorized as "Rare Diseases" [CONDITION-BROWSE-BRANCH]
- Trials that are labeled as interventional
- Study design: Randomized
  - Review trials individually to confirm
- Trial dates:
  - Registered (i.e. first received) January 1, 2010 through December 31, 2012
  - Registration date is no more than 60 days after start date
  - Discontinued or completed as of December 31, 2014
- Recruitment status listed as discontinued or completed (i.e. exclude if RECRUITMENT STATUS=’recruiting’, ‘enrolling by invitation’, ‘not yet recruiting’, ‘active not recruiting’)

**Definitions:**

- Discontinued trial: RECRUITMENT STATUS=’suspended’, ‘withdrawn’, and ‘terminated’
- Completed trial: RECRUITMENT STATUS=’completed’
- Rare disease: Listed as rare disease on GARD (Genetic and Rare Diseases Information Center <https://rarediseases.info.nih.gov/diseases>)
  - Perform manual review of all trials to ensure condition is a rare disease listed in the Genetic and Rare Disease (GARD) Information Center database
  - If trial includes more than one condition, more than half must meet definition of rare disease for the trial to be included
  - Infectious disease trials to be excluded given that these are often conducted in healthy volunteers for diseases that are rare in the US but not necessarily in other settings
- Funding source:
- Industry-funded=primary sponsor is industry
- Academia=primary sponsor university affiliate
- Government=funded by institutions such as the National Institutes of Health and other government entities outside of the United States
- Healthcare center=hospital, clinic, or clinical network as primary funder
- Other=all other funding sources
- Published: Publication as a peer-reviewed journal article

**Publication search:**

- Review ClinicalTrials.gov to find links to publications
- Search Medline via PubMed, Google Scholar and Embase
- If no publication identified, contact investigator via email
- All unpublished trials to be searched at end of study for “final search date”
- Publication needs to describe at least one of the primary trial end-points to be considered a trial publication

**Reasons for discontinuation:**

- Look for reasons listed in CT.gov; categorize as in Pica N, et al., 2016
- If no reason listed, or reason unclear, contact investigator via email to obtain information
- If >1 reason for discontinuation listed, use first listed reason

**Reasons to contact investigator:**

- For completed trials: no publications identified -> ask about publication
- For discontinued trials: no stated reason or reason unclear -> ask why discontinued

**Protocol for contacting investigators:**

- Identify email address in registry “responsible party” or in other publications by same investigator
- Send standard email with a reminder email 2 weeks later
- If no email address found or no response in 4 weeks, search for other email addresses online
- If these fail and there is a sponsoring agency, contact sponsor (e.g. pharmaceutical company)
- Contact investigators through company websites where applicable

**Analyses:**

- Prevalence of discontinuation
- Prevalence of non-publication at 2 and 4 years after completion
- Time to publication (Kaplan Meier curve)
- Reasons for discontinuation
- Characteristics of the different trial groups:
- Study population, i.e. adult vs pediatric population
- Conditions and interventions under study
- Funding type
- Trial characteristics, including sample size, masking, etc.
- Multivariable logistic regression to determine association of funding source with trial non-completion and time to publication
  - Control variables: intervention type, trial phase, masking, study population, sample size
